# Characterization and health risk assessment of PM_2.5_-bound polycyclic aromatic hydrocarbons in 5 urban cities of Zhejiang Province, China

**DOI:** 10.1038/s41598-019-43557-0

**Published:** 2019-05-13

**Authors:** Zhe Mo, Zhifang Wang, Guangming Mao, Xuejiao Pan, Lizhi Wu, Peiwei Xu, Shuchang Chen, Aihong Wang, Yongli Zhang, Jinbin Luo, Xialiang Ye, Xiaofeng Wang, Zhijian Chen, Xiaoming Lou

**Affiliations:** 1 Zhejiang Provincial Center for Disease Prevention and Control, Binsheng Road 3399, Hangzhou, 310051 Zhejiang China; 2 Hangzhou Center for Disease Prevention and Control, Mingshi Road 568, Hangzhou, Zhejiang P.R. China; 3 Ningbo Center for Disease Prevention and Control, Yongfeng Road 237, Ningbo, Zhejiang P.R. China; 4 Zhoushan Center for Disease Prevention and Control, Wengshan Road 568, Zhoushan, Zhejiang P.R. China; 5 Jinhua Center for Disease Prevention and Control, Jinou Road 1366, Jinhua, Zhejiang P.R. China; 6 Lishui Center for Disease Prevention and Control, Yushouerfu Road 28, Lishui, Zhejiang P.R. China

**Keywords:** Environmental impact, Risk factors

## Abstract

In 2015, we measured polycyclic aromatic hydrocarbons (PAHs) in atmospheric fine particulate matter (PM_2.5_) collected from 5 cities in Zhejiang Province. The mean toxic equivalent quotient (TEQ) values of benzo(a)pyrene (BaP) ranged between 1.2–3.1 ng/m^3^. The BaP-TEQ displayed seasonal trends, such that winter > spring and autumn > summer. During the winter, the most abundant individual PAHs were 4–6ring PAHs (84.04–91.65%). The median daily intake of atmospheric PAHs ranged between 2.0–7.4 ng/day for all populations, with seasonal trends identical to that of BaP-TEQ. The 95^th^ incremental lifetime cancer risk (ILCR) values induced by PM_2.5_-bound PAHs were far lower than 10^−6^ for all populations. The data suggested that the pollution levels in the 5 Zhejiang Province cities were higher than the Chinese National Ambient Air Quality Standard (NAAQS). In the future, relevant measures should be taken to control atmospheric PAHs, especially 4–6 ring PAHs. The data also revealed no obvious cancer risk for populations residing in these 5 cities of Zhejiang Province.

## Introduction

Polycyclic aromatic hydrocarbons (PAHs) are a large group of ubiquitous environmental poisons mainly sourced from the pyrolysis of organic matter or incomplete combustion^1^. Motor vehicles, home heating, fossil fuel combustion, and industrial processes are major sources of atmospheric PAHs^2^. After various combustion processes, gas phases or particulate-bound PAHs are emitted into the atmosphere^3^. The atmosphere is the most important means of PAHs dispersal^4^. Due to their widespread source and persistent characteristics, PAHs are dispersed through atmospheric transport and result in being ubiquitous in the environment^5^. Human beings are exposed to gas phases or particulate-bound PAHs in ambient air. Atmospheric PAHs adsorbed on fine particulate matter (PM_2.5_) enter into the human body through the respiratory system^6^. Several studies reported that 59–97% of atmospheric PAHs were adsorbed on PM_2.5_^7–10^. Therefore, recent studies have used atmospheric PAHs in PM_2.5_ to determine respiratory exposure concentrations^11–14^.

The U.S. Environmental Protection Agency has listed 16 PAHs as priority pollutants to regard to potential exposure and adverse health effects on humans^15^. Some PAHs are classified as carcinogenic materials to humans by the International Agency for Research on Cancer^16^. According to these, Benzo[a]pyrene was classified as carcinogenic material (Group 1), naphthalene, chrysene, benz[a]anthracene, benzo[k]fluoranthene and benzo[b]fluoranthene were classified as probably carcinogenic materials (Group 2B)^16^. Therefore, PAHs pollution poses a substantial concern.

China generates the highest PAHs emission^17^, at a total of 120,611 tons as of 2012^2^. There is a close correlation between human lung cancer and inhaled PAHs^18^. In recent years, lung cancer has become the 4^th^ leading cause of death in the Chinese population due to severe air pollution^19^. Three methods were applied for the carcinogenic risk assessment of PAHs: toxic equivalency factors (TEFs), the comparative potency of mixtures and the use of Benzo[a]pyrene as a surrogate^20,21^. A UK study found that the TEFs approach is more preferable than other two approaches for the estimate the carcinogenic risk associated with exposure to atmospheric PAHs^21^. Thus, we used TEFs method to assess the inhalation risk of PAHs to human health in our study.

Zhejiang Province is located in the Yangtze River Delta (YRD) region, which is considered one of the most rapidly developing regions of China. Severe atmospheric PAHs pollution has been detected in some regions of Zhejiang Province^22,23^, as well as in many Chinese cities experiencing urbanization and industrialization. To date, several cities in China have conducted health risk assessments of atmospheric PAHs^11,13,14,18,24–26^. However, most researchers focused on the risk of atmospheric PAHs by using a 24-hour exposure time, which could result in overestimations due to significant differences in outdoor and indoor PAHs concentrations^22^. Hence, we carried out an investigation to obtain an accurate exposure time. Research on the human health risk of PAHs via inhalation exposure is limited, regarding the Zhejiang Province.

The objectives of this study are to measure the concentration and distribution of atmospheric PAHs in PM_2.5_, to assess the daily intake of atmospheric PAHs in PM_2.5_ for different population groups, and to evaluate the lifetime carcinogenic risk of atmospheric PAHs in PM_2.5_ for different populations in Zhejiang Province.

## Results and Discussion

### Atmospheric PAHs in PM_2.5_

#### The concentrations of PM_2.5_

The concentrations of atmospheric PM_2.5_ in Hangzhou(HZ), Ningbo(NB), Jinhua (JH), Lishui (LS), and Zhoushan (ZS) were 79.4 ± 40.1, 63.0 ± 35.9, 61.7 ± 32.4, 49.0 ± 24.4, and 41.9 ± 31.6 µg/m^3^, respectively (Table S1). In all 5 cities, the concentration of PM_2.5_ exceeded the Chinese National Ambient Air Quality Standard (NAAQS) annual average PM_2.5_ value of 35 μg/m^3^ (GB 3095–2012)^27^.

#### The concentrations of Σ_16_ PAHs

**Figure 1:**
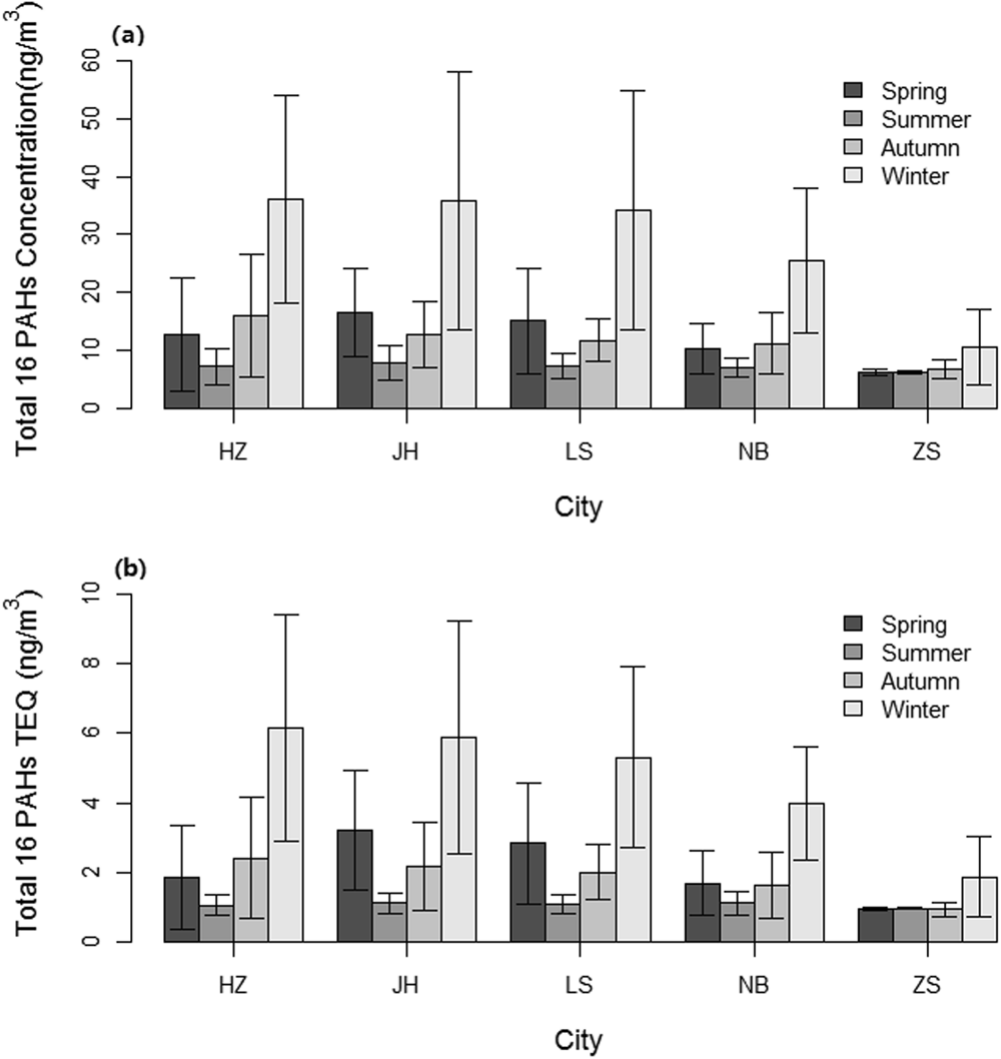
Concentration and TEQ of atmospheric PAHs in PM_2.5_ for different seasons by city: (**a**) Total of 16 PAH concentrations, (**b**) Total of 16 PAHs TEQ. The data are presented as the mean ± standard deviation. The figure was generated by R 64 3.3.1 with function *plot*: A Language and Environment for Statistical Computing, author: R Core Team, R Foundation for Statistical Computing, Vienna, Austria (https://www.R-project.org).

The concentrations of the sum of 16 atmospheric PAHs (Σ_16_ PAHs) in PM_2.5_ for the 5 selected cities in 2015 are shown in Table S1, Table S2, and Fig. 1. The concentrations of Σ_16_ PAHs in JH, HZ, LS, NB and ZS were 18.3 ± 16.0, 17.8 ± 15.8,16.9 ± 15.2, 13.5 ± 10.0, and 7.5 ± 4.0 ng/m^3^, respectively. For each city selected, the changing trends of Σ_16_ PAHs concentrations were similarly distributed by season, with a 1.7–5.0 times higher value in winter than summer. During the winter, the most abundant individual PAHs were 4–6 ring PAHs (84.0–91.7%). This result is in agreement with studies conducted in Taiyuan^13^ and other urban regions^14,28^. The potential reasons may be related with the more amount of incomplete combustion emissions from fossil fuel and other organic mass due to the residential heating, and lower levels of photochemical degradation in winter, as well as poor atmospheric diffusion in a certain meteorological condition with calm winds and temperature inversion during cold season^29,30^. Moreover, there is a smaller proportion of low molecular weight PAHs (2–3 ring) compared with 4–6 ring PAHs because there is a higher level of volatilization into the gaseous phase.

In winter, the concentrations of Σ_16_ PAHs in HZ, JH, and LS were 1.3–1.4 times higher than in NB, and 3.3–3.4 times higher than in ZS. Compared with the 1-year Σ_16_PAH concentrations in other areas of China, HZ, JH, and LS were similar to those in the Shenzhen suburbs (19.0 ng/m^3^ in PM_2.5_) of South China^14^, where our study area was also located. The Σ_16_ PAH concentrations in the 5 selected cities were 0.1–0.3 times that found in rural and urban Taiyuan (57.5–73.6 ng/m^3^ in PM_2.5_)^13^, and 0.01–0.8 times that observed in urban Tianjin in North China (23.4–513 ng/m^3^ in PM_10_)^29^. The latter location is a highly contaminated region due to a combination of high coal consumption and dense heavy traffic^13,29^. The decreasing trend of Σ_16_ PAH concentrations from North to South in China indicated spatial variation of the pollutants. This may be due to the different intensities of pollutant emission, geographical location, and adverse weather conditions^31^.

#### Potential Sources of PAHs

In our study, the ratio Fle/Pyr, BaP/BghiP, and BaP/(BaP + Chr) ranged from 0.75(LS) to 1.12(JH), 0.61(ZS) to 0.95(LS), and 0.47(NB) to 0.54(LS), respectively (Table S3). The ratios indicated that gasoline emissions may be the main source of PAHs in HZ, NB, and ZS, while the ratios in JH and LS suggested that gasoline emission and coal combustion both contributed.

#### Toxic equivalency quotient

The toxic equivalent quotient (TEQ) of the 5 selected cities is shown in Table S1 and Fig. 1. The TEQ in HZ, JH, LS, NB, and ZS were 2.8 ± 2.8, 3.1 ± 2.6, 2.8 ± 2.2, 2.1 ± 1.5, and 1.2 ± 0.7 ng/m^3^, respectively. Compared to the Chinese NAAQS (GB 3095-2012) annual average TEQ value for PAHs (1.0 ng/m^3^), the TEQs observed in HZ, JH, LS, NB, and ZS exceeded the standard. This finding was in accordance with the results of the PM_2.5_ concentrations. Thus, Σ_16_ PAHs may have negative effects in these cities.

During the study period, the TEQ displayed similar seasonal variation trends in each city. According to the seasonal distribution, the TEQ was listed, in descending order, as winter, spring and autumn, and summer. Coincidently, during the same study period, the seasonal trends of Σ_16_ PAH concentrations in each city were similar to those of the TEQ, due to a large proportion of 5–6 ring PAHs with high toxic equivalency factors (TEFs).

Compared to the 1-year TEQ in other areas of China, the TEQ in HZ, JH,LS, and NB were similar to those reported in China overall (2.4 ng/m^3^ based on high-resolution emission inventory)^24^ and in suburban Shenzhen (2.4 ng/m^3^ in PM_2.5_) in South China^14^, where our study area was also located. In the 5 studied cities, the TEQ is significantly lower than that reported in rural and urban Taiyuan (23.2 ± 30.8 and 27.4 ± 28.1 ng/m^3^ in PM_2.5_)^13^ and urban Tianjin (25.8 ng/m^3^ in PM_10_)^29^ in North China. The latter location is a highly contaminated region due to the spatial variation of pollutants, which is analysed as the Σ_16_ PAH concentrations. On the other hand, higher molecular weight PAHs with larger TEFs may be associated with vehicle emissions^29^, which suggests that vehicle combustion in North China may result in higher TEQs than observed in South China.

Overall, the 5 studied cities were only slightly polluted by PAHs when compared with other areas in China. However, the TEQs were all higher than the Chinese NAAQS. In the future, appropriate measures should be taken to control atmospheric PAHs, especially 4–6 ring PAHs, in Zhejiang.

### Daily intake exposure to atmospheric PAHs

**Figure 2:**
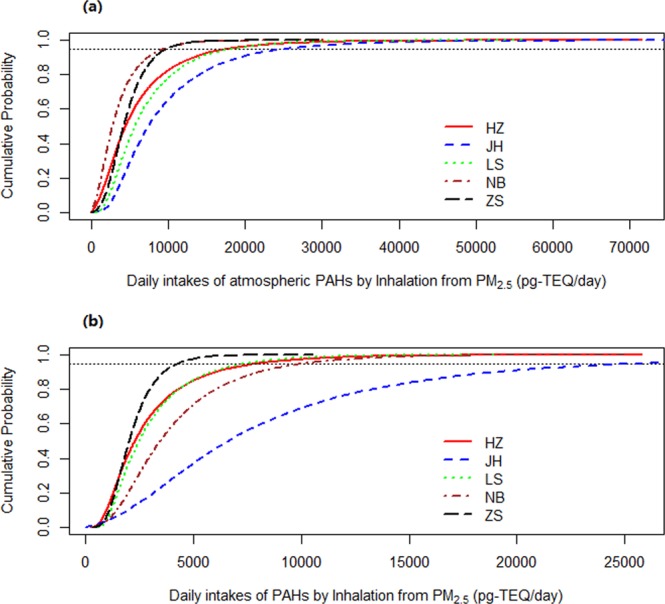
Probability distribution of daily intake exposure to atmospheric PAHs by inhalation from PM_2.5_ for different population groups in 5 selected cities of Zhejiang Province: (**a**) Adults, (**b**) Children. The figure was generated by R 64 3.3.1 with function *plot*: A Language and Environment for Statistical Computing, author: R Core Team, R Foundation for Statistical Computing, Vienna, Austria (https://www.R-project.org).

The cumulative probability distributions of the daily intake (DI) of PM_2.5_-bound Σ_16_PAHs for different population groups, over 4 seasons, in the 5 studied cities are shown in Table 1 and Figs 2, S1–S3. The median DI for adults in JH, LS, HZ, ZS, and NB were 7.4, 5.6, 4.4, 4.2, and 2.8 ng/day, which was basically in line with the ranking of mean of TEQ (Table S1), for children in JH, NB, LS, HZ, ZS as the decreasing order were 6.6, 3.5, 2.5, 2.3 and 2.0 ng/day, which was completely in line with the ranking of mean of exposure time (Table S4).

**Table 1:** Estimated daily intakes of atmospheric PAHs by inhalation from PM_2.5_ via Monte Carlo method^a^ (HZ: Hangzhou, JH: Jinhua, LS: Lishui, NB: Ningbo, ZS: Zhoushan).

City	Population	Gender	Spring	Summer	Autumn	Winter	Annual
HZ	Adults	Male	4.2 (4.2)	2.7 (2.4)	4.9 (5.5)	12.9 (13.5)	5.5 (6.8)
		Female	2.7 (2.5)	1.7 (1.4)	3.1 (3.2)	8.2 (8.0)	3.5 (4.1)
		Total	3.5 (3.5)	2.2 (1.9)	4.0 (4.4)	10.6 (11.0)	4.4 (5.5)
	Children	Male	1.7 (1.3)	1.1 (0.6)	2.0 (1.8)	5.3 (4.2)	2.3 (2.3)
		Female	1.8 (1.3)	1.1 (0.6)	2.1 (1.8)	5.5 (4.1)	2.3 (2.3)
		Total	1.7 (1.3)	1.1 (0.6)	2.0 (1.8)	5.4 (4.1)	2.3 (2.3)
JH	Adults	Male	8.9 (7.7)	3.9 (2.1)	6.5 (5.2)	17.7 (13.5)	8.4 (8.8)
		Female	6.9 (5.7)	3.0 (1.4)	5.0 (3.7)	13.4 (9.6)	6.5 (6.5)
		Total	7.9 (6.9)	3.5 (1.8)	5.7 (4.5)	15.5 (11.8)	7.4 (7.8)
	Children	Male	7.1 (7.6)	3.2 (2.5)	5.2 (5.2)	14.2 (13.8)	6.7 (8.1)
		Female	6.8 (7.0)	3.1 (2.3)	5.0 (4.8)	13.6 (12.6)	6.4 (7.5)
		Total	7.0 (7.4)	3.2 (2.4)	5.2 (5.1)	13.9 (13.5)	6.6 (7.9)
LS	Adults	Male	6.6 (6.1)	3.2 (1.8)	5.6 (3.7)	14.2 (10.0)	6.4 (6.4)
		Female	5.1 (4.5)	2.4 (1.3)	4.3 (2.7)	10.8 (7.3)	4.9 (4.8)
		Total	5.8 (5.4)	2.8 (1.6)	4.9 (3.2)	12.4 (8.7)	5.6 (5.7)
	Children	Male	2.6 (2.1)	1.2 (0.5)	2.1 (1.1)	5.4 (3.1)	2.5 (2.3)
		Female	2.5 (2.0)	1.2 (0.4)	2.1 (1.0)	5.3 (2.9)	2.5 (2.2)
		Total	2.6 (2.1)	1.2 (0.4)	2.1 (1.0)	5.4 (3.0)	2.5 (2.2)
NB	Adults	Male	3.1 (3.1)	2.3 (2.1)	2.9 (3.0)	6.3 (6.8)	3.4 (3.9)
		Female	1.9 (1.9)	1.4 (1.2)	1.8 (1.8)	4.0 (4.2)	2.1 (2.4)
		Total	2.5 (2.5)	1.8 (1.7)	2.3 (2.4)	5.2 (5.5)	2.8 (3.2)
	Children	Male	3.2 (2.3)	2.3 (1.4)	2.9 (2.3)	6.6 (5.4)	3.5 (3.3)
		Female	3.1 (2.2)	2.3 (1.3)	2.9 (2.2)	6.5 (5.0)	3.4 (3.1)
		Total	3.1 (2.3)	2.3 (1.4)	2.9 (2.2)	6.6 (5.3)	3.5 (3.2)
ZS	Adults	Male	4.6 (2.7)	4.5 (2.6)	4.1 (2.7)	7.8 (6.5)	5.0 (3.9)
		Female	3.2 (1.7)	3.2 (1.7)	2.9 (1.8)	5.5 (4.4)	3.5 (2.6)
		Total	3.9 (2.2)	3.8 (2.2)	3.5 (2.3)	6.6 (5.5)	4.2 (3.3)
	Children	Male	1.9 (0.7)	1.8 (0.7)	1.7 (0.8)	3.2 (2.3)	2.0 (1.3)
		Female	1.8 (0.7)	1.8 (0.7)	1.6 (0.8)	3.2 (2.2)	2.0 (1.3)
		Total	1.8 (0.7)	1.8 (0.7)	1.7 (0.8)	3.2 (2.2)	2.0 (1.3)

^a^Estimated daily intakes (Median, with inter-quartile range in ng/day) of atmospheric PAHs resulting from inhalation of PM2.5. Estimates were calculated using the Monte Carlo method.

In the spring, the median DI was estimated to be 2.5–7.9 ng/day for adults and 1.7–7.0 ng/day for children. During summer, the median DI was approximately 1.8–3.8 ng/day for adults and 1.1–3.2 ng/day for children. In autumn, the median DI was 2.3–5.7 ng/day for adults and 1.7–5.2 ng/day for children. During winter, the median DI was 5.2–15.5 ng/day for adults and 3.2–13.9 ng/day for children (Table 1). According to the seasonal distribution, the DI was listed, in descending order, as winter, spring and autumn, and summer for both adults and children (Table 1 and Figs S1, S2). This result was in accordance with the TEQ ranking due to the large contribution of TEQ to DI when the constant effects of inhalation rate and exposure time are considered for different seasons.

According to the population, adults have higher DI levels than children (Table 1 and Fig. S3) owing to the fact that inhalation rate ratios were higher than exposure time ratios between children and adults (Table S4). However, NB is the exception to this observation, with an inhalation rate ratio (1.8) that was less than the exposure time ratio (2.5) between children and adults (Tables 1, S4 and Fig. S3). The result of the former was in line with that reported in Taiyuan^13^, while the latter was agreed with that reported in Shenzhen^14^. Children in HZ had an inhalation rate ratio (1.02) that was slightly less than the exposure time ratio (1.03). Males showed a slightly higher exposure dose than females in all cities for both adults and children because the male inhalation rates and exposure times were higher than females in all adults and in children from JH, LS, and ZS (Tables 1, S4 and Fig. S3). Although the exposure time of male children in NB was less than that of female children, the inhalation exposure of the male children was higher (Table S4), resulting in a higher DI in male children (Table 1 and Fig. S3). The result of the former was in line with that reported in Shenzhen^14^, while the latter was in line with that reported in Taiyuan^13^.

However, our results were far lower than that in previous reports conducted in Taiyuan (outdoors for adolescents, adults, and seniors: 104–307 ng/day)^13^, Tianjin (outdoors for children:321.6 ng/day; adults: 519 ng/day)^11^, Taiwan (rural, industrial, and high traffic for infants: 252 ng/day; children: 1590 ng/day; adults:1628 ng/day)^18^. The main reason for this difference could be that those reports assume that the daily exposure time for the population was 1 day, whereas most people may not remain outdoors for an entire day. The exposure time in the present study was 1.9–7.9 hours (approximately 0.1–0.3 of 1 day), which was calculated based on population surveys. The difference between the DI of PAHs given the real exposure time and 1-day exposure time was shown in Table S5. The TEQ values in the present study were also lower than those previously reported (except Shenzhen)^14^.

### Incremental lifetime cancer risk

**Figure 3:**
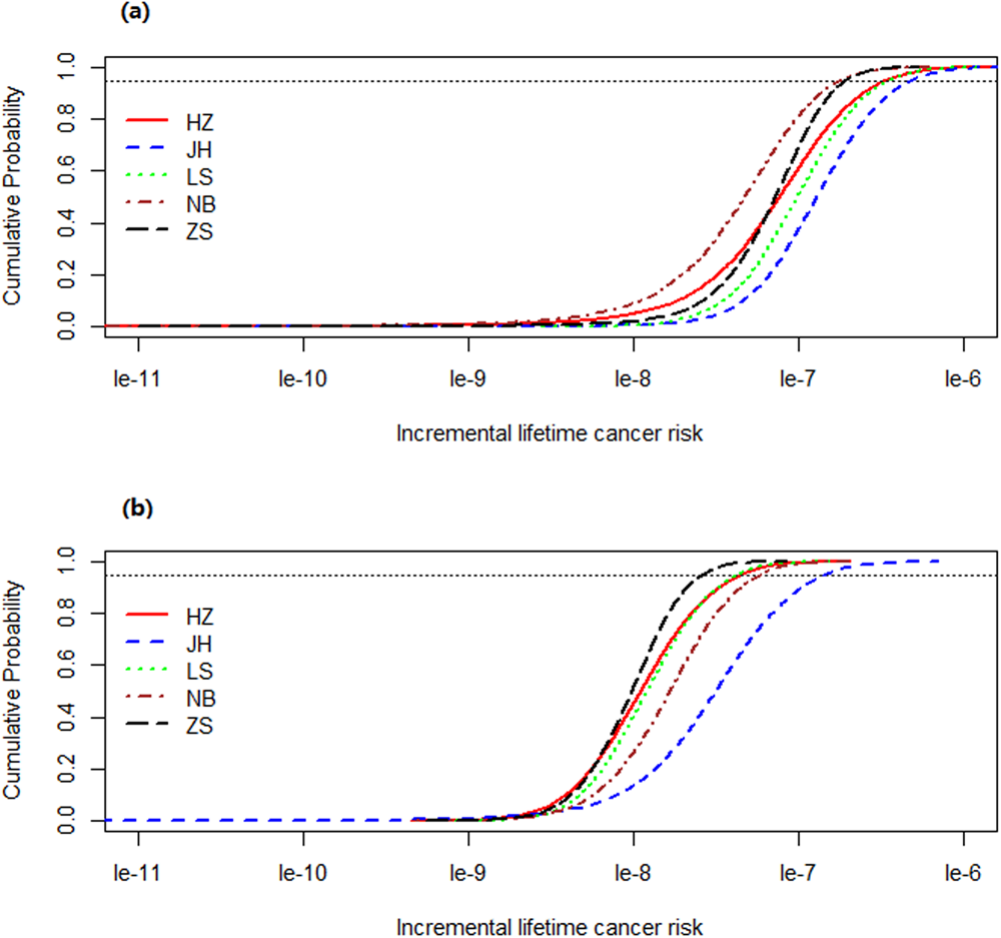
Cumulative probability distribution of incremental lifetime cancer risk induced by atmospheric PAHs via inhalation from PM_2.5_ for different population groups in 5 selected cities of Zhejiang Province: (**a**) Adults, (**b**) Children. The figure was generated by R 64 3.3.1 with function *plot*: A Language and Environment for Statistical Computing, author: R Core Team, R Foundation for Statistical Computing, Vienna, Austria (https://www.R-project.org).

Under most regulatory programs, an incremental lifetime cancer risk (ILCR) less than 10^−6^ indicates acceptable risk, between 10^−6^–10^−4^ designates a potential risk, and greater than 10^−4^ suggests a public health concern^13,32^. The cumulative probability distributions of the ILCR induced by atmospheric PAHs are shown in Table 2 and Figs 3 and S4.

**Table 2:** 95^th^incremental lifetime cancer risk induced by atmospheric PAHs by inhalation from PM_2.5_ via Monte Carlo method (HZ: Hangzhou, JH: Jinhua, LS: Lishui, NB: Ningbo, ZS: Zhoushan).

City	Adults			Children		
	Male	Female	Total	Male	Female	Total
HZ	4.1 × 10^−7^	2.7 × 10^−7^	3.4 × 10^−7^	4.6 × 10^−8^	4.2 × 10^−8^	4.5 × 10^−8^
JH	5.4 × 10^−7^	4.3 × 10^−7^	4.8 × 10^−7^	1.5 × 10^−7^	1.3 × 10^−7^	1.5 × 10^−7^
LS	3.9 × 10^−7^	3.1 × 10^−7^	3.5 × 10^−7^	4.4 × 10^−8^	3.9 × 10^−8^	4.3 × 10^−8^
NB	2.1 × 10^−7^	1.4 × 10^−7^	1.8 × 10^−7^	6.0 × 10^−8^	5.4 × 10^−8^	5.9 × 10^−8^
ZS	2.2 × 10^−7^	1.6 × 10^−7^	1.9 × 10^−7^	2.7 × 10^−8^	2.4 × 10^−8^	2.6 × 10^−8^

For adults, the 95^th^ ILCR induced by PM_2.5_-bound Σ_16_ PAHs in JH, LS, HZ, ZS, and NB were 4.8 × 10^−7^, 3.5 × 10^−7^, 3.4 × 10^−7^, 1.9 × 10^−7^, and 1.8 × 10^−7^ (Table 2 and Fig. 3), which was in line with the ranking of DI, for children in JH, NB, HZ, LS, ZS as the decreasing order were 1.5 × 10^−7^, 5.9 × 10^−8^, 4.5 × 10^−8^, 4.3 × 10^−8^ and 2.6 × 10^−8^ (Table 2 and Fig. 3), which was also basically in line with the ranking of DI.

**Figure 4:**
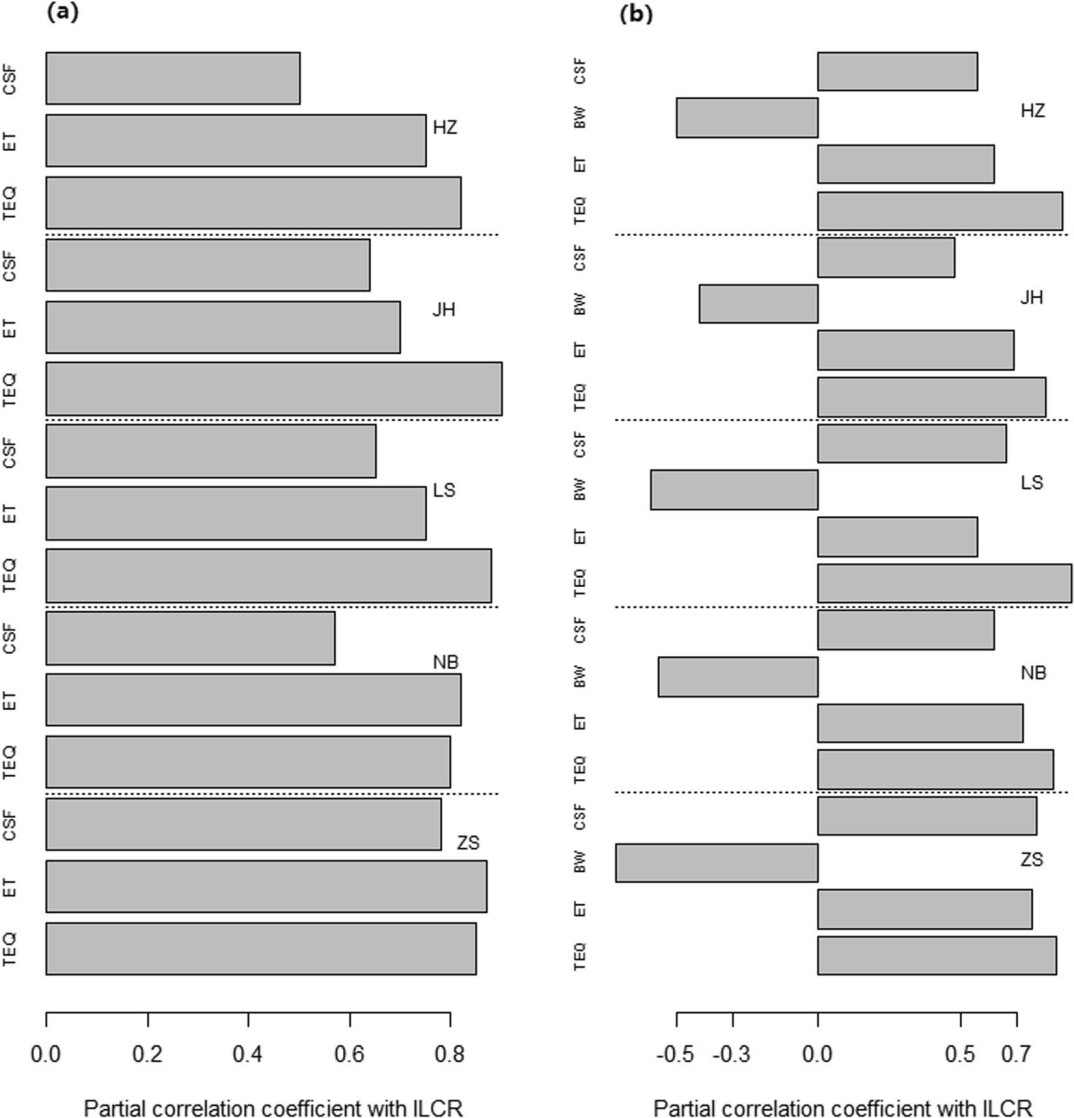
Sensitivity analysis results for the incremental lifetime cancer risk assessment in 5 cities of Zhejiang Province: (**a**) Adults, (**b**) Children. The figure was generated by R 64 3.3.1 with function *plot*: A Language and Environment for Statistical Computing, author: R Core Team, R Foundation for Statistical Computing, Vienna, Austria (https://www.R-project.org).

According to the population, the 95^th^ ILCR were estimated to be 2.1 × 10^−7^–5.4 × 10^−7^ for adult males, 1.4 × 10^−7^–4.3 × 10^−7^ for adult females, 2.7 × 10^−8^–1.5 × 10^−7^ for male children, and 2.4 × 10^−8^–1.3 × 10^−7^ for female children. The ILCR, in descending order, was listed as adult males, adult females, male children, and female children (Table 2 and Fig. 4). These results may be attributed to the higher level of DI exposure to atmospheric PAHs in the adult and male populations, as well as the lower exposure duration in children despite their lower body weights (Table S4). This result was in accordance with a study conducted in Shenzhen^14^.

Similar to the DI results, the ILCR values were far lower than the mean determined by previous reports in Taiyuan (children: 1.7 × 10^−6^, adults: 8.0 × 10^−6^)^13^, Taiwan (children: 6.5 × 10^−6^, adults: 1.0 × 10^−4^)^18^, and Tianjin (2.7 × 10^−4^)^11^. Though the TEQ in our study was similar to that in Shenzhen, the ILCR values were 0.01–0.3 times the level in Shenzhen (2.0 × 10^−7^–1.3 × 10^−6^)^14^. This is mainly due to overexposure in the daily outdoor exposure time, which is analysed in the DI exposure to atmospheric PAHs section. Those values were far higher than that in Guangzhou (children: 2.2 × 10^−11^, adults: 6.7 × 10^−11^)^33^.

Overall, the ILCR values were all less than 10^−6^ for adult males, adult females, male children, and female children. These results suggested that no obvious cancer risk existed for populations living in the 5 selected cities of Zhejiang Province.

### Sensitivity and uncertain analysis

The results of the sensitivity analysis on the ILCR for different population groups in the 5 selected cities are shown in Table S6 and Fig. 4. For the adult population, the partial correlation coefficient with ILCR was listed, in descending order, as TEQ (0.8–0.9), exposure time (ET) (0.7–0.9), and cancer slope factor (CSF) (0.5–0.8). For the child population, the partial correlation coefficient with ILCR was listed, in descending order, as TEQ (0.8–0.9), ET (0.5–0.8), CSF (0.5–0.8), and body weight (BW) (−0.7 – −0.4). Based on the sensitivity results, TEQ and ET are the 2 most influential variables in the health risk assessments of both children and adults. Therefore, improving the accuracy of ET and TEQ could enhance the accuracy of health risk assessments.

**Figure 5:**
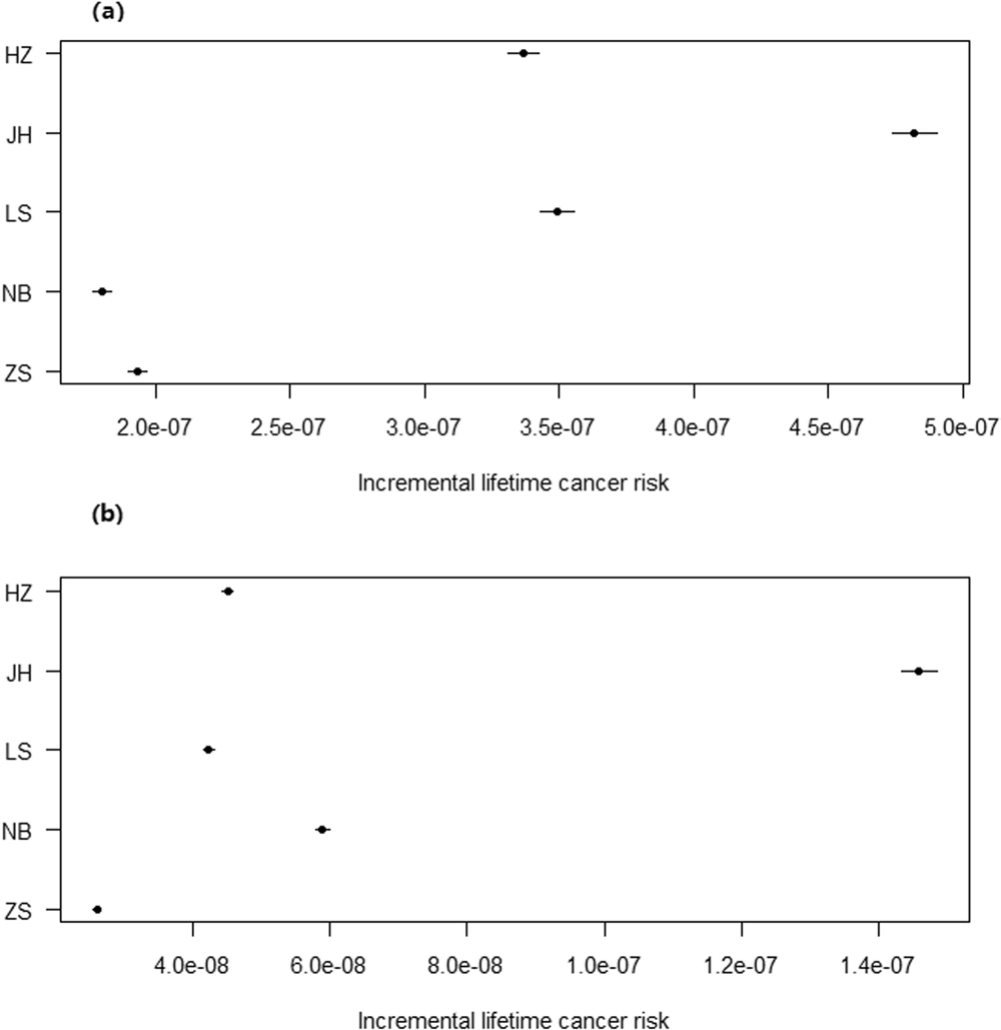
Uncertainty analysis results for the incremental lifetime cancer risk assessment of the cancer slope factor in 5 cities of Zhejiang Province: (**a**) Adults, (**b**) Children. The figure was generated by R 64 3.3.1 with function *plot*: A Language and Environment for Statistical Computing, author: R Core Team, R Foundation for Statistical Computing, Vienna, Austria (https://www.R-project.org).

Based on the modelling of the distribution parameter uncertainty results, Table S7 and Fig. 5 show the results of the 95% confidence interval with an ILCR of 100 values. We can see that the uncertainty in the “max” parameter has a small effect on the risk distribution for this scenario due to the narrower range of the 95% confidence interval for the 95^th^ ILCR (Table S7 and Fig. 5). These data indicated that we could neglect the effect from the missing max values of CSF.

### Uncertainty and limitation of health risk assessments

In this study, only 1 sampling site was set up in each city’s traditional centre district. The results of the health risk assessment would be more representative, if more sampling sites were built in different regions, such as industrial or high traffic regions.

Although the atmospheric PAH concentrations in PM_2.5_ were detected for 7 days/month in this study, the day-to-day variations in the concentration of atmospheric PAHs in PM_2.5_ could lead to some uncertainties due to meteorological conditions such as temperature, wind speed, and humidity^34^.

Moreover, the mean time spent outdoors was investigated as 2.1 ± 1.7 to 5.9 ± 2.6 hours/day and 2.9 ± 0.7 to 7.0 ± 4.1 hours/day for adults and children, respectively. Hence, people likely spend most of their time indoors. In general, the concentration of atmospheric PAHs in PM_2.5_ were very different between outdoors and in doors^22^. In our study, only outdoor contaminants were used to calculate the exposure dose and ILCR; indoor contaminants were not taken into consideration. Furthermore, atmospheric PAHs in the gaseous phase were not considered in our study. These limitations would lead to a possible bias that partially underestimates the exposure dose and ILCR. Although there are some uncertainties and limitations, this study still provides a valuable evaluation of health risks associated with exposure to atmospheric PAHs in PM_2.5_.

## Conclusions

Our data suggested that the pollution levels, in the 5 selected cities of Zhejiang Province, were higher than the Chinese NAAQS. In the future, appropriate measures should be taken to control atmospheric PAHs, especially 4–6 ring PAHs. The pollution level and inhalation exposure of atmospheric PAHs in PM_2.5_ were listed, in descending order, as winter, spring and autumn, and summer. The ILCR values were far lower than 10^−6^, which suggested no obvious cancer risk for populations residing in the 5 selected cities of Zhejiang Province. Due to the aforementioned limitations, future studies should consider indoor pollutant data and sample more sites in different regions.

## Materials and Methods

### Site description

**Figure 6:**
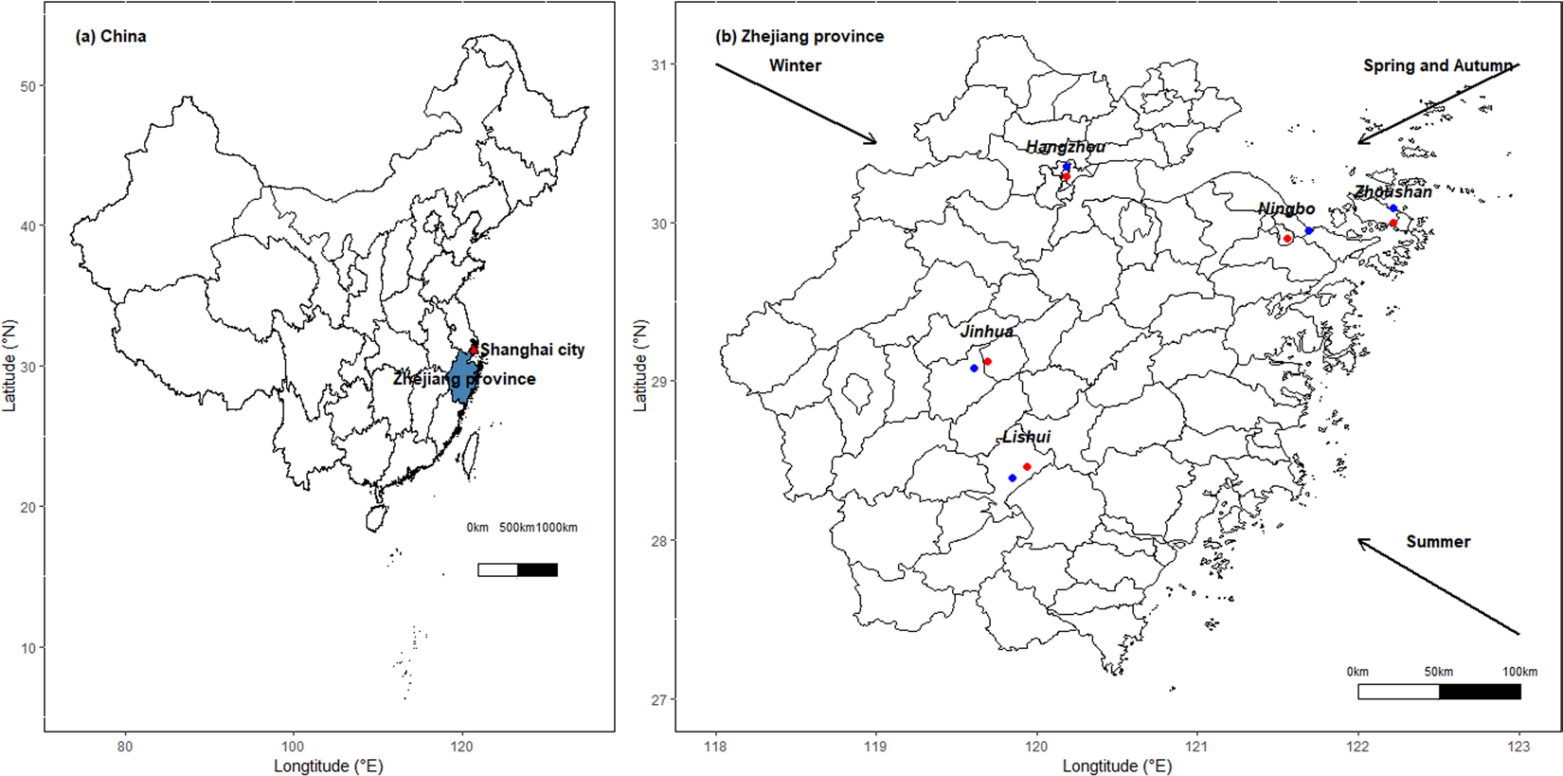
Location of the survey areas in Zhejiang Province, China: (**a**) The Zhejiang province in China; (**b**) The sampling sites in each city: the red point is sampling site of each city; the blue point is major industrial area of each city; the arrow is dominant winds trajectories of each season. The figure was generated by R 64 3.3.1: A Language and Environment for Statistical Computing, author: R Core Team, R Foundation for Statistical Computing, Vienna, Austria (https://www.R-project.org), with Package *ggplot2* version 2.2.1 (https://cran.r-project.org/web/packages/ggplot2/index.html).

This study was conducted in Zhejiang Province (between 118°E–123°E and 27.12°N –31.31°N), an eastern coastal region of China, which covers 11cities. The province features a subtropical climate with 4 distinct seasons. In this study, cluster sampling was applied to select 5 traditional urban centres with populations greater than 10,000 in Zhejiang Province (Hangzhou (HZ), Jinhua (JH), Lishui (LS), Ningbo (NB), and Zhoushan (ZS)). One PM_2.5_ sampling site was set up in each selected community centre (Fig. 6). Detailed information on sampling sites is in Table S8.

HZ is the capital of Zhejiang Province and is a famous tourist city with more than 2.6 million vehicles. NB is the world’s 4^th^ largest port city and is an important chemical industry base in Zhejiang Province. ZS is an island city, which is the largest seafood production, processing, and marketing base in China. JH is located in the middle of Zhejiang Province and is known for the manufacture of metal products and medicine. LS is mainly in mountainous and hilly terrain. In winter, electricity and natural gas was the main heating situations in these 5 selected cities.

### Sampling

A total of 485 samples were collected for 23 h (starting at 9:00 a.m. local time each day and ending at 8:00 a.m. the next day) from the 10^th^ to 16^th^between January and December 2015. The sampling sites were set up on rooftops in each traditional centre district; the sites were established 12–15 m above ground to avoid airflow obstacles. Atmospheric PM_2.5_ samples were collected on 47-mm glass fibre filters (QZ47DMCAN, MTL, America) using a mid-volume sampler (MVS6, LECKEL, Germany) at a constant flow rate of 38.3 L/min. The samples were weighed by an automated filter weighing system (WZZ-02, Weizhizhao, Hangzhou, China) before and after sampling to determine the mass of PM_2.5_. All the filters were transported immediately to the laboratory in a filter holder and stored at −20 °C until analysis. Meteorological parameters, such as temperature and wind speed, were also recorded at the time of sample collection. Detailed information on the temperature and wind speed is in Table S8.

### Extraction and analysis of PAHs

The filter membrane sample was cut into pieces and placed into a 15-mL centrifuge tube. Meanwhile, 0.2 μg/mL decafluorobiphenyl was added as internal standard. Ultrasonic extraction was applied for 10 min, and the sample was centrifuged at a speed of 8000 r/min for 5 min. Finally, the extracts were filtered prior to separation with a 0.45 μm membrane (Agilent) and stored in a refrigerator until analysis^3^.

PAHs, including naphthalene (Nap), acenaphthylene (Acy), fluorene (Flu), acenaphthene (Ace), phenanthrene (Phe), anthracene (Ant), fluoranthene (Fle), pyrene (Pyr), chrysene (Chr), benz(a)anthracene (BaA), benzo(b)fluoranthene (BbF), benzo(k)fluoranthene (BkF), benzo(a)pyrene (BaP), dibenz(a,h)anthracene (DBA), benzo(g,h,i)perylene (BghiP), and indeno(1,2,3-cd)pyrene (InP), were determined using high performance liquid chromatography (HPLC) with fluorescence and UV detectors (Agilent 1260). A 4.60 mm × 250 mm, 3.5 μm C_18_ reversed-phase column (Agilent) was used; the column temperature was 35 °C and the mobile phase flow rate was 0.8 mL/min. The gradient elution program was the following: 72% acetonitrile and 28% water were added and held for 27 min; 100% acetonitrile was added at a rate of 2.5% acetonitrile/min until the peak was achieved. The HPLC system was calibrated using an external standard. A standard reference material (O2Si, standard PAH mixture) soluble in acetonitrile was used at different dilutions to obtain calibration curves for each run. The concentration range of the calibration standards were 0, 0.05, 0.1, 0.5, 1.0, and 5.0 μg/mL. A good agreement existed between standard and sample chromatograms obtained on a given day. Since the fluorescence detector was unable to detect all 16 PAHs, we used fluorescence and UV detectors to determine the PAHs simultaneously^3^. The detection wavelengths of fluorescence and UV detectors were set at the values given in Table S9. QA/QC included reagent blanks, analytical duplicates, and analysis of the standard reference material (O2Si, standard PAH mixture). The detection limits of PAHs corresponding to the fluorescence and UV detectors are shown in Table S10. The relative standard deviation was less than 10%. The PAHs recovery percentage of 16 PAHs from the standard material was between 65% and 96% (Table S10). The method we used to determine the PAHs was based on the Chinese National Ambient Air Quality Standard (NAAQS)^35^.

### Population survey

Exposure time in outdoor environments is an important parameter for health risk assessment. Approximately 1000 adults (>18 years old) and 600 school children (9–11 years old) were randomly selected from each community to conduct an exposure time investigation by questionnaire. Exposure time showed significant regional and individual differences.

In the study, systematic sampling was applied to select households from each community based on the household size. All family members greater than 18 years old were recruited. One primary school from each community was selected by the cluster sampling method. In each school, children aged 9 to 11 years were randomly selected as subjects. Subjects were introduced to the study protocol.

The first part of the questionnaire involved demographic characteristics, including age, gender, and occupation. The second part of the questionnaire was divided into 2 periods: rest and working days, including indoor and outdoor time at home, work (for adults), school (for children), travel, and other activities. Indoor and outdoor time at work was not included in the rest days of adults. The time in the questionnaires was accurate to the minute, and the investigation was carried out by well-trained public health doctors. Eventually, the average daily outdoor exposure time was obtained. Detailed information on the exposure time questionnaire is in the supplementary information. The weight measurement was performed using the World Health Organization’s (WHO) standard methods^36^, which require the child to take off their shoes, belt, and wear light clothing. Weight measurements were precise to 0.1 kg. Detailed results for exposure time and weight are in Table S4.

### Data analysis

#### Toxic equivalency quotient

The carcinogenic effect of mixtures of atmospheric PAHs was expressed as the TEQ of BaP. The TEQ of atmospheric PAHs in PM_2.5_ were calculated according to formula (1):

1 $${\rm{TEQ}}=\sum _{{\rm{i}}={\rm{1}}}^{n}{C}_{i}\times {{\rm{TEF}}}_{i}$$

where Ci = concentration of PAHs congener i (pg/m^3^); TEF*i* = Toxic equivalency Factor of PAHs congener i^37^ (Table S1).

#### Daily intake estimates

The DI of atmospheric PAHs in PM_2.5_ for different populations during 4 seasons was calculated according to formula (2):

2 $${\rm{DI}}=({\rm{TEQ}}\times {\rm{IR}}\times {\rm{ET}})/24$$

where TEQ = the toxic equivalent quotient of BaP (pg/m^3^); IR = Inhalation rate (m^3^/day); ET = Exposure times (hours/day); unit of 24 is hours/day (Table S4). The cumulative probability of DI is calculated by the Monte Carlo simulation method running 10,000 iterations in which TEQ obeys lognormal distribution and ET obeys normal distribution (R 3.3.1 software with EnvStats packages version 2.1.0).

#### Incremental lifetime cancer risk

ILCR was calculated according to formula (3):

3 $${\rm{ILCR}}=({\rm{TEQ}}\times {\rm{IR}}\times {\rm{CF}}\times {\rm{ED}}\times {\rm{ET}}\times {\rm{EF}})\times {\rm{CSF}}/({\rm{BW}}\times {\rm{AT}})$$

where TEQ = the toxic equivalent quotient of BaP (pg/m^3^); IR = Inhalation rate(m^3^/d); CF = Conversion factor (mg/pg); ED = Exposure duration (years); ET = Exposure times (hours/day); EF = Exposure frequency (days/year); CSF = Cancer slope factor (mg/kg-day)^−1^, which was regarded as 3.14(1.80) (Geometric Mean (Geometric Standard Deviation))^18^; BW = Body weight (kg); AT = Averaging time (hours). Detailed information is presented in Table S4. The cumulative probability of ILCR is calculated by the Monte Carlo simulation method running 10,000 iterations in which TEQ obeys lognormal distribution, ET obeys normal distribution, CSF obeys lognormal distribution, and BW (for children) obeys normal distribution (R 3.3.1 software with EnvStats packages vision 2.1.0).

#### Potential Sources of PAHs

Atmosphere PAHs are mainly derived from incomplete combustion of fuels such as gasoline, diesel and coal. Due to different fuel type and combustion conditions, the amount and range of PAHs produced from any pyrolytic process varies widely^38^. Therefore, the potential source of PAHs could be determined based on the characteristic ratio of PAHs. The ratios of Fle/Pyr, BaP/BghiP, and BaP/(BaP + Chr) are employed as diagnostic tools to identify the potential dominant sources of PAHs in ambient air due to the factor that the values are different to various sources such as gasoline, diesel and coal combustion, respectively^39–42^ (Table S3).

#### Sensitivity and uncertain analysis

The partial correlation coefficient between input variables (TEQ, ET, CSF for adults, TEQ, ET, BW, and CSF for children) and the output variable (ILCR) was used to determine the sensitivity of the input variable^43,44^. Since the max values of input variable, CSF, was not given in Chen *et al*.^18^, we used the value of log (Geometric Mean) + 3 × log (Geometric Standard Deviation) to replace the max value using uncertainty quantification based modelling of distribution parameter uncertainty^43,44^. In brief, we simulated the max value 100 times, assuming a uniform distribution ranging from 2 to 4 times the standard deviation in the uncertainty analysis. The 2-dimensional Monte Carlo analysis (2-D MCA) was performed by first generating 100 random “max” parameter values of the truncated lognormal distribution for CSF using Latin hypercube sampling (100 iterations of the outer loop representing uncertainty). Then, for each of these 100 values, 10,000 iterations of the inner loop (representing variability) were run to calculate the 95% confidence interval for the 95^th^ ILCR (Table S11). The sensitivity and uncertainty analyses were conducted based on the EnvStats packages^43,44^.

#### Definition of variables

Seasons were categorized into 4 groups: March, April, and May were defined as spring; June, July, and August were defined as summer; September, October, and November were defined as autumn; and December, January, and February were defined as winter. Since lifetime cancer risk was regarded as the lifetime exposure risk, seasonal differences in cancer risk were not analysed in this study. Based on age and gender, populations were categorized into 4 groups: male children, female children, male adults, and female adults. All calculations, plotting, and statistical analysis were performed using R 3.3.1 software.

#### Ethical approval and informed consent

All research protocols involved in this study were approved by the Ethics Committee of Zhejiang Provincial Center for Disease Control and Prevention. All methods were carried out in accordance with the relative guidelines and regulations. Written informed consents were obtained from all participants or, if subjects are under 18, from a parent and/or legal guardian prior to enrolment.

## Supplementary information

Characterization and health risk assessment of PM2.5-bound polycyclic aromatic hydrocarbons in 5 urban cities of Zhejiang Province, China
