# Supplementary material for: Characterization and health risk assessment of PM2.5-bound polycyclic aromatic hydrocarbons in 5 urban cities of Zhejiang Province, China
Source: Sci Rep. 2019 May 13;9:7296. doi: 10.1038/s41598-019-43557-0 (PMC6513845; doi:10.1038/s41598-019-43557-0)
Supplement: Supplementary file 1 — Characterization and health risk assessment of PM2.5-bound polycyclic aromatic hydrocarbons in 5 urban cities of Zhejiang Province, China [file 41598_2019_43557_MOESM1_ESM.doc]

# Characterization and health risk assessment of PM_2.5_-bound polycyclic aromatic hydrocarbons in 5 urban cities of Zhejiang Province, China

Zhe Mo ^1^,Zhifang Wang ^1^, Guangming Mao ^1^, Xuejiao Pan ^1^, Lizhi Wu ^1^, Peiwei Xu ^1^, Shuchang Chen^2^, Aihong Wang ^3^,Yongli Zhang^4^, Jinbin Luo ^5^, Xialiang Ye ^6^,Xiaofeng Wang^1^, Zhijian Chen ^1^, Xiaoming Lou^1^

^1^ Zhejiang Provincial Center for Disease Prevention and Control, Binsheng Road 3399, Hangzhou 310051, Zhejiang, China

^2^ Hangzhou Center for Disease Prevention and Control, Mingshi Road 568, Hangzhou, Zhejiang, P.R. China

^3^ Ningbo Center for Disease Prevention and Control, Yongfeng Road 237, Ningbo, Zhejiang, P.R. China

^4^ Zhoushan Center for Disease Prevention and Control, Wengshan Road 568, Zhoushan, Zhejiang, P.R. China

^5^ Jinhua Center for Disease Prevention and Control, Jinou Road 1366, Jinhua, Zhejiang, P.R. China

^6^ Lishui Center for Disease Prevention and Control, Yushouerfu Road 28, Lishui, Zhejiang, P.R. China

Correspondence: Xiaoming Lou, Department of Environmental and Occupational Health, Zhejiang Provincial Center for Disease Control and Prevention, Binsheng Road 3399#, Hangzhou 310051, Zhejiang Province, China; [xmlou@cdc.zj.cn](mailto:xmlou@cdc.zj.cn). 0086057187115219.

Co-correspondence: Xiaofeng Wang, Department of Environmental and Occupational Health, Zhejiang Provincial Center for Disease Control and Prevention, Binsheng Road 3399#, Hangzhou 310051, Zhejiang Province, China; xfwang@cdc.zj.cn. 008657187115222.

Co-correspondence: Zhijian Chen, Department of Environmental and Occupational Health, Zhejiang Provincial Center for Disease Control and Prevention, Binsheng Road 3399#, Hangzhou 310051, Zhejiang Province, China; [zhjchen@cdc.zj.cn](mailto:zhjchen@cdc.zj.cn). 008657187115226.

**Figures**

**
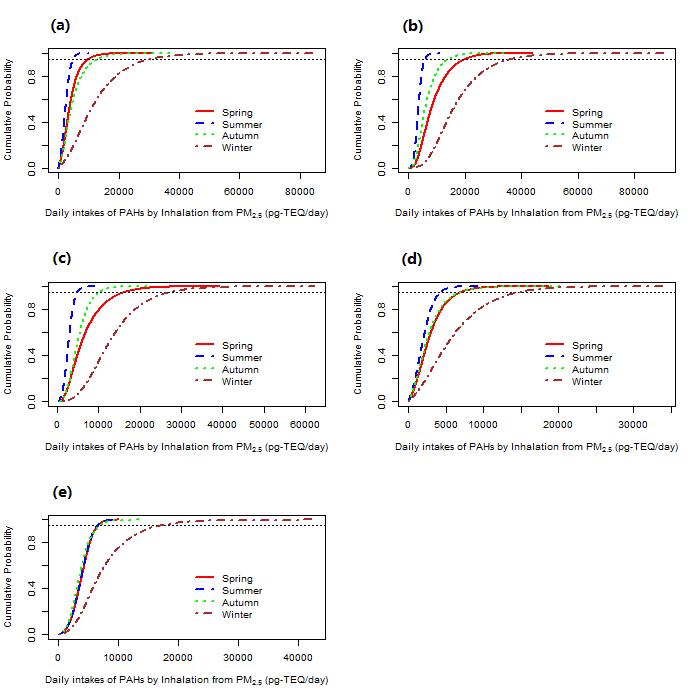
**

**Figure S1.** Probability distribution of daily intake exposure to atmospheric PAHs by inhalation from PM_2.5_ samples for different season in adults: (a) HZ, (b) JH, (c) LS, (d) NB, and (e) ZS.

**
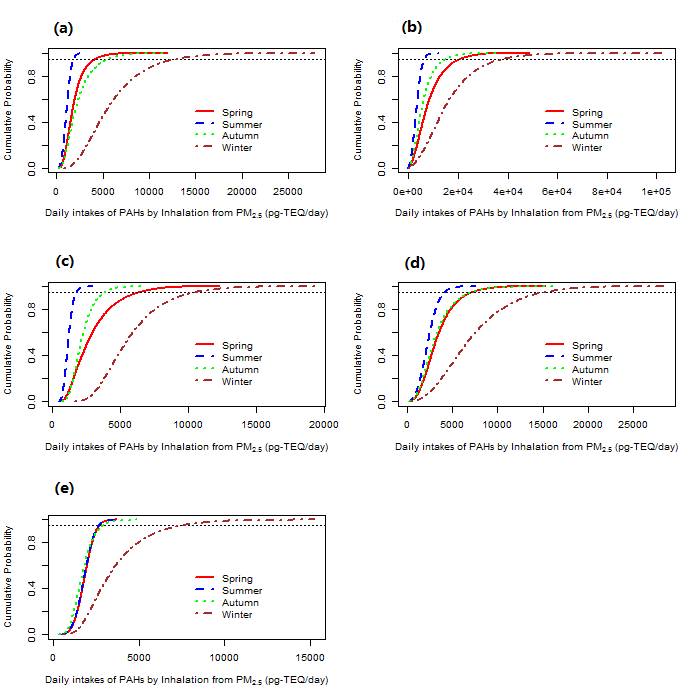
 Figure S2.** Probability distribution of daily intake exposure to atmospheric PAHs by inhalation from PM_2.5_ for different season in children: (a) HZ, (b) JH, (c) LS, (d) NB, and (e) ZS.

**
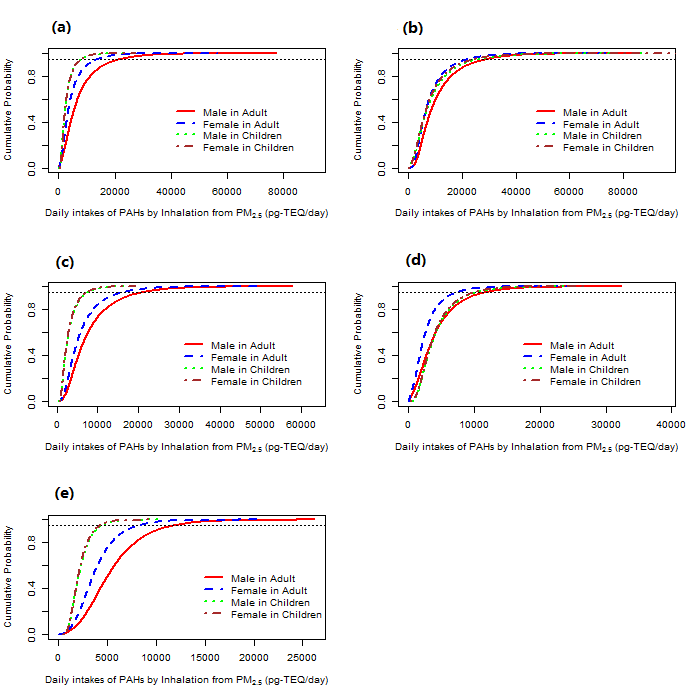
**

**Figure S3.** Probability distribution of daily intake exposure to atmospheric PAHs by inhalation from PM_2.5_ for different gender groups: (a) HZ, (b) JH, (c) LS, (d) NB, and (e) ZS.

**
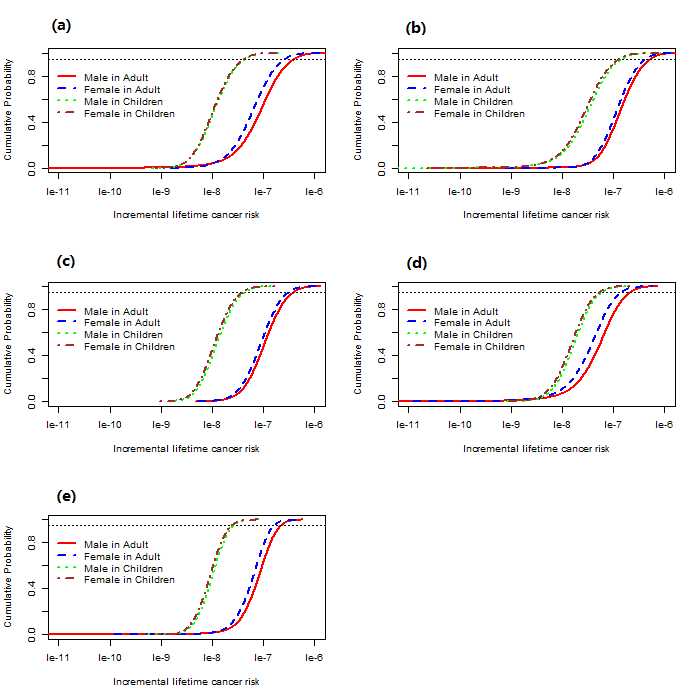
**

**Figure S4.** Cumulative probability distribution of Incremental lifetime cancer risk induced by atmospheric PAHs by inhalation from PM_2.5_ for different gender groups: (a) HZ, (b) JH, (c) LS, (d) NB, and (e) ZS.

**Tables**

**Table.S1 Estimated concentration and TEQ of atmospheric PAHs in PM_2.5_ by city (HZ: Hangzhou, JH: Jinhua, LS: Lishui, NB: Ningbo, ZS: Zhoushan)**

| Compounds | Abbreviation | TEF | HZ | | JH | | LS | | NB | | ZS | |
| --- | --- | --- | --- | --- | --- | --- | --- | --- | --- | --- | --- | --- |
|  |  |  | Concentration ^a^ | TEQ ^b^ | Concentration ^a^ | TEQ ^b^ | Concentration ^a^ | TEQ ^b^ | Concentration ^a^ | TEQ ^b^ | Concentration ^a^ | TEQ ^b^ |
| Naphthalene | Nap | 0.001 | 315.39±134.04 | 0.32±0.13 | 354.23±277.59 | 0.35±0.28 | 336.53±160.02 | 0.34±0.16 | 341.40±194.09 | 0.34±0.19 | 281.25±58.6 | 0.28±0.06 |
| Acenaphthylene | Acy | 0.001 | 470.53±105.06 | 0.47±0.11 | 476.42±127.65 | 0.48±0.13 | 475.58±145.78 | 0.48±0.15 | 465.59±112.77 | 0.47±0.11 | 462.5±117.20 | 0.46±0.12 |
| Fluorene | Flu | 0.001 | 285.26±52.53 | 0.29±0.05 | 284.91±53.08 | 0.28±0.05 | 308.42±104.84 | 0.31±0.10 | 282.80±56.38 | 0.28±0.06 | 281.25±58.60 | 0.28±0.06 |
| Acenaphthene | Ace | 0.001 | 285.26±52.53 | 0.29±0.05 | 284.91±53.08 | 0.28±0.05 | 308.42±104.84 | 0.31±0.10 | 282.80±56.38 | 0.28±0.06 | 281.25±58.60 | 0.28±0.06 |
| Phenanthrene | Phe | 0.001 | 624.32±876.99 | 0.62±0.88 | 525.14±661.94 | 0.53±0.66 | 607.05±762.61 | 0.61±0.76 | 454.69±552.93 | 0.45±0.55 | 284.89±69.58 | 0.28±0.07 |
| Anthracene | Ant | 0.01 | 567.16±950.72 | 5.67±9.51 | 508.68±416.86 | 5.09±4.17 | 780.11±1387.79 | 7.8±13.88 | 465.59±112.77 | 4.66±1.13 | 462.50±117.20 | 4.63±1.17 |
| Fluoranthene | Fle | 0.001 | 1110.59±1409.13 | 1.11±1.41 | 1296.93±1795.18 | 1.30±1.80 | 1076.95±1323.07 | 1.08±1.32 | 982.86±1052.47 | 0.98±1.05 | 525.09±317.67 | 0.53±0.32 |
| Pyrene | Pyr | 0.001 | 1249.86±1674.75 | 1.25±1.67 | 1156.51±1342.88 | 1.16±1.34 | 1441.58±1717.15 | 1.44±1.72 | 1258.82±1602.13 | 1.26±1.60 | 590.54±480.46 | 0.59±0.48 |
| Chrysene | Chr | 0.01 | 1477.73±1756.33 | 14.78±17.56 | 1400.33±1773.34 | 14.00±17.73 | 1167.37±1397.05 | 11.67±13.97 | 995.35±1140.29 | 9.95±11.4 | 456.15±497.56 | 4.56±4.98 |
| Benz(a)anthracene | BaA | 0.1 | 958.12±1192.64 | 95.81±119.26 | 1066.64±1475.81 | 106.66±147.58 | 993.05±1247.58 | 99.31±124.76 | 647.47±699.47 | 64.75±69.95 | 352.77±244.28 | 35.28±24.43 |
| Benzo(b)fluoranthene | BbF | 0.1 | 4141.56±4294.70 | 414.16±429.47 | 4189.24±4343.55 | 418.92±434.35 | 3436.84±3709.52 | 343.68±370.95 | 2623.11±2663.21 | 262.31±266.32 | 941.46±1569.82 | 94.15±156.98 |
| Benzo(k)fluoranthene | BkF | 0.1 | 1029.97±1189.52 | 103.00±118.95 | 938.06±937.55 | 93.81±93.76 | 866.32±815.51 | 86.63±81.55 | 664.80±593.18 | 66.48±59.32 | 357.17±244.81 | 35.72±24.48 |
| Benzo(a)pyrene | BaP | 1 | 1252.03±1464.46 | 1252.03±1464.46 | 1483.56±1651.23 | 1483.56±1651.23 | 1375.79±1440.54 | 1375.79±1440.54 | 884.06±889.79 | 884.06±889.79 | 420.59±370.77 | 420.59±370.77 |
| Dibenz(a,h)anthracene | DBA | 1 | 739.00±824.45 | 739.00±824.45 | 776.48±693.25 | 776.48±693.25 | 669.26±478.54 | 669.26±478.54 | 663.38±527.19 | 663.38±527.19 | 534.71±225.73 | 534.71±225.73 |
| Benzo(g,h,i)perylene | BghiP | 0.01 | 1517.11±1678.95 | 15.17±16.79 | 1620.47±1666.31 | 16.20±16.66 | 1442.32±1252.66 | 14.42±12.53 | 1154.30±1444.05 | 11.54±14.44 | 685.41±601.49 | 6.85±6.01 |
| Indeno(1,2,3-cd) pyrene | InP | 0.1 | 1806.32±1858.13 | 180.63±185.81 | 1897.49±1773.69 | 189.75±177.37 | 1589.58±1413.09 | 158.96±141.31 | 1319.22±1220.75 | 131.92±122.07 | 587.19±628.62 | 58.72±62.86 |
| - | Σ16PAHs | - | 17830.20±15774.55 | 2824.59±2761.71 | 18259.98±16015.87 | 3108.85±2643.77 | 16875.16±15200.08 | 2772.08±2236.93 | 13486.23±10038.00 | 2103.12±1530.52 | 7504.71±3970.41 | 1197.91±741.47 |
| - | PM_2.5_ ^c^ | - | 79.35±40.08 | - | 61.72±32.43 | - | 48.99±24.44 | - | 62.98±35.86 | - | 41.88±31.62 | - |

^a^ Mean ± Standard deviation, unit: pg/m^3^ ; ^b^ Mean ± Standard deviation, unit: pg /m^3^; ^c^ unit: μg/m^3^

**Table.S2** Estimated mean concentration of atmospheric PAHs in PM_2.5_ by city in winter and summer (ng/m^3^) (HZ: Hangzhou, JH: Jinhua, LS: Lishui, NB: Ningbo, ZS: Zhoushan)

| Compounds | Aromatic ring | HZ | | JH | | LS | | NB | | ZS | |
| --- | --- | --- | --- | --- | --- | --- | --- | --- | --- | --- | --- |
|  |  | Summer | Winter | Summer | Winter | Summer | Winter | Summer | Winter | Summer | Winter |
| Nap | 2 | 0.30 | 0.35 | 0.30 | 0.45 | 0.30 | 0.35 | 0.30 | 0.35 | 0.30 | 0.23 |
| Acy | 3 | 0.50 | 0.38 | 0.53 | 0.38 | 0.54 | 0.36 | 0.50 | 0.36 | 0.50 | 0.37 |
| Flu | 3 | 0.30 | 0.24 | 0.30 | 0.24 | 0.30 | 0.23 | 0.30 | 0.23 | 0.30 | 0.23 |
| Ace | 3 | 0.30 | 0.24 | 0.30 | 0.24 | 0.30 | 0.23 | 0.30 | 0.23 | 0.30 | 0.23 |
| Phe | 3 | 0.30 | 1.56 | 0.30 | 1.16 | 0.30 | 1.41 | 0.30 | 0.87 | 0.30 | 0.25 |
| Ant | 3 | 0.50 | 0.78 | 0.50 | 0.54 | 0.50 | 1.66 | 0.50 | 0.36 | 0.50 | 0.37 |
| Fle | 4 | 0.49 | 2.84 | 0.57 | 3.34 | 0.50 | 2.70 | 0.50 | 2.39 | 0.50 | 0.59 |
| Pyr | 4 | 0.50 | 3.25 | 0.57 | 2.45 | 0.50 | 3.81 | 0.50 | 3.28 | 0.50 | 0.76 |
| Chr | 4 | 0.32 | 3.80 | 0.33 | 3.51 | 0.32 | 2.91 | 0.32 | 2.63 | 0.30 | 0.86 |
| BaA | 4 | 0.32 | 2.50 | 0.30 | 2.79 | 0.29 | 2.50 | 0.30 | 1.60 | 0.30 | 0.52 |
| BbF | 5 | 0.91 | 6.48 | 1.36 | 7.65 | 1.03 | 6.55 | 0.91 | 4.27 | 0.44 | 2.02 |
| BkF | 5 | 0.61 | 1.94 | 0.35 | 1.91 | 0.31 | 1.73 | 0.34 | 1.29 | 0.30 | 0.52 |
| BaP | 5 | 0.30 | 2.99 | 0.32 | 3.20 | 0.32 | 2.92 | 0.32 | 2.02 | 0.30 | 0.72 |
| InP | 5 | 0.45 | 3.90 | 0.60 | 3.85 | 0.55 | 3.27 | 0.47 | 2.65 | 0.30 | 1.25 |
| DBA | 6 | 0.50 | 1.59 | 0.50 | 0.98 | 0.50 | 0.90 | 0.56 | 0.94 | 0.50 | 0.68 |
| BghiP | 6 | 0.60 | 3.35 | 0.69 | 3.25 | 0.65 | 2.75 | 0.57 | 2.10 | 0.50 | 0.93 |
| Σ PAHs _4-6 rings_ |  | 5.00 | 32.64 | 5.60 | 32.91 | 4.97 | 30.04 | 4.79 | 23.16 | 3.94 | 8.85 |
| Σ_16_PAHs |  | 7.20 | 36.18 | 7.83 | 35.91 | 7.21 | 34.27 | 6.99 | 25.56 | 6.14 | 10.53 |
| Σ PAHs _4-6 rings_/Σ_16_PAHs (%) | | 69.45 | 90.21 | 71.55 | 91.65 | 68.98 | 87.65 | 68.52 | 90.62 | 64.18 | 84.04 |

**Table.S3 Diagnostic ratios of PAHs and those of reported sources (HZ: Hangzhou, JH: Jinhua, LS: Lishui, NB: Ningbo, ZS: Zhoushan)**

| City | Fle/Pyr | BaP/BghiP | BaP/(BaP+Chr) |
| --- | --- | --- | --- |
| HZ | 0.89 | 0.83 | 0.46 |
| JH | 1.12 | 0.92 | 0.51 |
| LS | 0.75 | 0.95 | 0.54 |
| NB | 0.78 | 0.77 | 0.47 |
| ZS | 0.89 | 0.61 | 0.48 |
| Gasoline engine | <1.0[^1^](#_ENREF_1) | 0.30-0.44[^2^](#_ENREF_2) | 0.49[^3^](#_ENREF_3) |
| Diesel engine | <1.0[^1^](#_ENREF_1) | 0.30-0.44[^2^](#_ENREF_2) | 0.76[^3^](#_ENREF_3) |
| Coal combustion | 1.0-1.4[^1^](#_ENREF_1) | 0.9-6.6[^2^](#_ENREF_2) | 0.07-0.24[^4^](#_ENREF_4) |

**Table.S4** Parameters used in the risk assessment (HZ: Hangzhou, JH: Jinhua, LS: Lishui, NB: Ningbo, ZS: Zhoushan)

窗体顶端

| Variable | Units | Distribution ^a^ | Adults | | | Children | | | References ^a^ |
| --- | --- | --- | --- | --- | --- | --- | --- | --- | --- |
|  |  |  | Male | Female | Total | Male | Female | Total |  |
| Body weight (BW) |  | Point estimate/Normal |  |  |  |  |  |  | CEFH[^5^](#_ENREF_5) /This study |
| HZ | kg |  | 65.00 | 56.00 | 60.60 | 36.01±8.95 | 33.05±7.92 | 34.57±8.59 |  |
| JH | kg |  | 65.00 | 56.00 | 60.60 | 35.03±10.17 | 33.25±10.15 | 34.23±10.20 |  |
| LS | kg |  | 65.00 | 56.00 | 60.60 | 34.86±8.94 | 32.85±13.97 | 33.76±12.00 |  |
| NB | kg |  | 65.00 | 56.00 | 60.60 | 36.83±11.11 | 34.61±10.27 | 35.71±10.74 |  |
| ZS | kg |  | 65.00 | 56.00 | 60.60 | 36.19±9.46 | 33.87±9.07 | 34.98±9.32 |  |
| Averaging time (AT) | hours | Point estimate | 70×365×24 | 70×365×24 | 70×365×24 | 70×365×24 | 70×365×24 | 70×365×24 |  |
| Conversion factor (CF) | mg/ng | Point estimate | 10^-6^ | 10^-6^ | 10^-6^ | 10^-6^ | 10^-6^ | 10^-6^ | *-* |
| Exposure duration (ED) | years | Point estimate | 43.00 | 43.00 | 43.00 | 7.00 | 7.00 | 7.00 | Xia[^6^](#_ENREF_6) |
| Exposure frequency (EF) | days/year | Point estimate | 365.00 | 365.00 | 365.00 | 365.00 | 365.00 | 365.00 |  |
| Exposure times (ET) |  | Normal |  |  |  |  |  |  | This study |
| HZ | hours/day |  | 3.02±2.31 | 2.45±1.59 | 2.76±2.03 | 2.63±1.00 | 2.71±1.00 | 2.67±1.00 |  |
| JH | hours/day |  | 4.59±1.66 | 4.32±1.38 | 4.46±1.53 | 7.10±4.42 | 7.02±4.07 | 7.06±4.27 |  |
| LS | hours/day |  | 3.79±1.60 | 3.59±1.42 | 3.69±1.52 | 2.89±0.72 | 2.86±0.66 | 2.88±0.69 |  |
| NB | hours/day |  | 2.33±1.93 | 1.86±1.42 | 2.11±1.72 | 5.22±2.23 | 5.26±2.09 | 5.24±2.16 |  |
| ZS | hours/day |  | 6.27±2.76 | 5.46±2.21 | 5.88±2.55 | 5.11±1.49 | 5.08±1.37 | 5.09±1.43 |  |
| Inhalation rate (IR) | m^3^/day | Point estimate | 17.70 | 14.30 | 16.00 | 8.93 | 8.76 | 8.85 | CEFH[^5^](#_ENREF_5)/Xia[^6^](#_ENREF_6) |
| Cancer slope factor (CSF) | (mg/kg-day)^-1^ | Lognormal | 3.14±1.80 | 3.14±1.80 | 3.14±1.80 | 3.14±1.80 | 3.14±1.80 | 3.14±1.80 | Chen[^3^](#_ENREF_3) |

^a^ The former reference of “/” is for adults, the latter reference of “/” is for children.

**Table.S5** The difference between daily intakes of PAH between real exposure time and whole day exposure time (ng/day) (HZ: Hangzhou, JH: Jinhua, LS: Lishui, NB: Ningbo, ZS: Zhoushan)

| City | Adults | Children |
| --- | --- | --- |
| HZ | 34.1 | 18.2 |
| JH | 32.6 | 15.9 |
| LS | 31.0 | 18.2 |
| NB | 28.5 | 12.4 |
| ZS | 12.9 | 7.5 |

**Table.S6** Sensitivity analysis results on the incremental lifetime cancer risk assessment (HZ: Hangzhou, JH: Jinhua, LS: Lishui, NB: Ningbo, ZS: Zhoushan)

| City | Gender | Adults ^a^ | | |  | Children ^a^ | | | |
| --- | --- | --- | --- | --- | --- | --- | --- | --- | --- |
|  |  | TEQ | ET | CSF |  | TEQ | ET | BW | CSF |
| HZ | Male | 0.81 | 0.75 | 0.49 |  | 0.87 | 0.64 | -0.47 | 0.58 |
|  | Female | 0.83 | 0.73 | 0.52 |  | 0.86 | 0.60 | -0.53 | 0.57 |
|  | Total | 0.82 | 0.75 | 0.50 |  | 0.86 | 0.62 | -0.50 | 0.56 |
| JH | Male | 0.89 | 0.71 | 0.63 |  | 0.81 | 0.70 | -0.38 | 0.48 |
|  | Female | 0.90 | 0.69 | 0.65 |  | 0.80 | 0.67 | -0.44 | 0.47 |
|  | Total | 0.90 | 0.70 | 0.64 |  | 0.80 | 0.69 | -0.42 | 0.48 |
| LS | Male | 0.88 | 0.76 | 0.64 |  | 0.90 | 0.59 | -0.57 | 0.68 |
|  | Female | 0.88 | 0.74 | 0.65 |  | 0.89 | 0.54 | -0.62 | 0.65 |
|  | Total | 0.88 | 0.75 | 0.65 |  | 0.89 | 0.56 | -0.59 | 0.66 |
| NB | Male | 0.80 | 0.82 | 0.57 |  | 0.84 | 0.74 | -0.53 | 0.64 |
|  | Female | 0.80 | 0.81 | 0.58 |  | 0.83 | 0.71 | -0.58 | 0.62 |
|  | Total | 0.80 | 0.82 | 0.57 |  | 0.83 | 0.72 | -0.56 | 0.62 |
| ZS | Male | 0.85 | 0.87 | 0.77 |  | 0.86 | 0.77 | -0.68 | 0.78 |
|  | Female | 0.86 | 0.87 | 0.79 |  | 0.84 | 0.73 | -0.73 | 0.76 |
|  | Total | 0.85 | 0.87 | 0.78 |  | 0.84 | 0.75 | -0.71 | 0.77 |

^a^ Partial correlation coefficient with ILCR

**Table.S7** Uncertain analysis results on the incremental lifetime cancer risk assessment for Cancer slope factor in five cities of Zhejiang province (HZ: Hangzhou, JH: Jinhua, LS: Lishui, NB: Ningbo, ZS: Zhoushan)

| Population | Mean ^a^ | Sderr ^b^ | Lower limit of C.I ^c^ | Upper limit of C.I ^d^ |
| --- | --- | --- | --- | --- |
| Adults in HZ | 3.37×10^-7^ | 2.85×10^-8^ | 3.42×10^-7^ | 3.31×10^-7^ |
| Adults in JH | 4.82×10^-7^ | 4.18×10^-8^ | 4.90×10^-7^ | 4.74×10^-7^ |
| Adults in LS | 3.49×10^-7^ | 3.15×10^-8^ | 3.55×10^-7^ | 3.43×10^-7^ |
| Adults in NB | 1.80×10^-7^ | 1.66×10^-8^ | 1.83×10^-7^ | 1.77×10^-7^ |
| Adults in ZS | 1.93×10^-7^ | 1.77×10^-8^ | 1.97×10^-7^ | 1.90×10^-7^ |
| Children in HZ | 4.52×10^-8^ | 4.06×10^-9^ | 4.60×10^-8^ | 4.44×10^-8^ |
| Children in JH | 1.46×10^-7^ | 1.30×10^-8^ | 1.48×10^-7^ | 1.43×10^-7^ |
| Children in LS | 4.24×10^-8^ | 3.76×10^-9^ | 4.31×10^-8^ | 4.16×10^-8^ |
| Children in NB | 5.90×10^-8^ | 5.32×10^-9^ | 6.00×10^-8^ | 5.79×10^-8^ |
| Children in ZS | 2.61×10^-8^ | 2.42×10^-9^ | 2.66×10^-8^ | 2.56×10^-8^ |

^a^ incremental lifetime cancer risk Mean

^b^ incremental lifetime cancer risk Standard error

^c^ incremental lifetime cancer risk Lower limit of confident interval

^d^ incremental lifetime cancer risk Upper limit of confident interval

**Table.S8 Information on sampling sites (HZ: Hangzhou, JH: Jinhua, LS: Lishui, NB: Ningbo, ZS: Zhoushan)**

| City | District | Community | School | Longitude ^a^ (°E) | Latitude ^a^ (°N) | Elevation ^a^ (m) | Temperature ^b^ (℃) | Wind speed ^b^ (m/s) |
| --- | --- | --- | --- | --- | --- | --- | --- | --- |
| HZ | Xiacheng | Yugongqiao | Modern Experimental Primary School | 120.18 | 30.29 | 14 | 17.03±7.71 | 1.99±0.75 |
| JH | Wucheng | Yangguang | Dongguan Primary School | 119.69 | 29.12 | 41 | 17.98±7.75 | 1.63±0.50 |
| LS | Liandu | Tianning | Liandu Primary School | 119.94 | 28.46 | 61 | 18.47±7.41 | 0.94±0.37 |
| NB | Jiangbei | Baisha | Jiangbei District Experimental Primary School | 121.56 | 29.9 | 9 | 16.87±8.45 | 1.92±0.80 |
| ZS | Dinghai | Lincheng | Zhoushan First Primary School | 122.22 | 30 | 7 | 16.20±7.73 | 1.87±0.84 |

^a^ The geographic information on PM_2.5_ sampling sites; ^b^ Mean ± Standard deviation

**Table.S9** The wavelength of PAHs corresponding to Fluorescence and UV detectors

窗体底端

| ID | PAHs | Abbreviation | Excitation wavelength\emission wavelength  with Fluorescence detector (nm) | Absorption wavelength  with UV detector (nm) |
| --- | --- | --- | --- | --- |
| 1 | Naphthalene | Nap | 280/324 | 220 |
| 2 | Acenaphthylene | Acy | - | 230 |
| 3 | Fluorene | Flu | 280/324 | 254 |
| 4 | Acenaphthene | Ace | 280/324 | 230 |
| 5 | Phenanthrene | Phe | 254/350 | 254 |
| 6 | Anthracene | Ant | 254/400 | 254 |
| 7 | Fluoranthene | Fle | 290/460 | 230 |
| 8 | Pyrene | Pyr | 336/376 | 230 |
| 9 | Chrysene | Chr | 275/385 | 254 |
| 10 | Benz(a)anthracene | BaA | 275/385 | 290 |
| 11 | Benzo(b)fluoranthene | BbF | 305/430 | 254 |
| 12 | Benzo(k)fluoranthene | BkF | 305/430 | 290 |
| 13 | Benzo(a)pyrene | BaP | 305/430 | 290 |
| 14 | Dibenz(a,h)anthracene | DBA | 305/430 | 290 |
| 15 | Benzo(g,h,i)perylene | BghiP | 305/430 | 220 |
| 16 | Indeno(1,2,3-cd) pyrene | InP | 305/500 | 254 |

**Table.S10 The detection limits and recovery percentage of PAHs corresponding to Fluorescence and UV detectors**

| ID | PAHs | Abbreviation | Detection limit with Fluorescence detector (ng/m^3^) | Detection limit with UV detector (ng/m^3^) | Recovery percentage (%) |
| --- | --- | --- | --- | --- | --- |
| 1 | Naphthalene | Nap | 0.26 | 0.26 | 65.8 |
| 2 | Acenaphthylene | Acy | - | 0.13 | 74.9 |
| 3 | Fluorene | Flu | 0.06 | 0.13 | 85.4 |
| 4 | Acenaphthene | Ace | 0.10 | 0.13 | 85.9 |
| 5 | Phenanthrene | Phe | 0.12 | 0.14 | 78.0 |
| 6 | Anthracene | Ant | 0.10 | 0.10 | 86.1 |
| 7 | Fluoranthene | Fle | 0.07 | 0.14 | 87.7 |
| 8 | Pyrene | Pyr | 0.10 | 0.10 | 90.8 |
| 9 | Chrysene | Chr | 0.06 | 0.10 | 92.7 |
| 10 | Benz(a)anthracene | BaA | 0.07 | 0.12 | 92.1 |
| 11 | Benzo(b)fluoranthene | BbF | 0.05 | 0.14 | 95.1 |
| 12 | Benzo(k)fluoranthene | BkF | 0.06 | 0.12 | 96.0 |
| 13 | Benzo(a)pyrene | BaP | 0.04 | 0.14 | 87.2 |
| 14 | Dibenz(a,h)anthracene | DBA | 0.05 | 0.07 | 94.3 |
| 15 | Benzo(g,h,i)perylene | BghiP | 0.09 | 0.13 | 94.9 |
| 16 | Indeno(1,2,3-cd) pyrene | InP | 0.07 | 0.13 | 94.5 |

**Table.S11 Uncertain analysis on Cancer slope factor**

| Variable | Description | 2-Dimensions Monte Carlo analysis |
| --- | --- | --- |
| CSF | Cancer slope factor (mg/kg-day)^-1^ | Lognormal Geometric mean (GeoMean) =3.14 Geometric Standard deviation (GeoStd) =1.80  Max ~ uniform Min =log(GeoMean) + 2× log(GeoStd) Max = log(GeoMean) + 4×log(GeoStd) |

**Exposure time questionnaire for adult**

1. Gender: 1) Male 2) Female

2. Age: _____

3. Occupation: _____

4. Working day:

4.1 Total time at home: __ hour__ minute

4.1.1 Indoor time: __ hour__ minute

4.1.2 Outdoor time: __ hour__ minute

4.2 Total time at work: __ hour__ minute

4.2.1 Indoor time: __ hour__ minute

4.2.2 Outdoor time: __ hour__ minute

4.3 Total time at travel: __ hour__ minute

4.3.1 Indoor time: __ hour__ minute

4.3.2 Outdoor time: __ hour__ minute

4.4 Total time at other situations: __ hour__ minute

4.4.1 Indoor time: __ hour__ minute

4.4.2 Outdoor time: __ hour__ minute

5. Rest day:

5.1 Total time at home: __ hour__ minute

5.1.1 Indoor time: __ hour__ minute

5.1.2 Outdoor time: __ hour__ minute

5.2 Total time at travel: __ hour__ minute

5.2.1 Indoor time: __ hour__ minute

5.2.2 Outdoor time: __ hour__ minute

5.3 Total time at other situations: __ hour__ minute

5.3.1 Indoor time: __ hour__ minute

5.3.2 Outdoor time: __ hour__ minute

**Exposure time questionnaire for children**

1. Gender: 1) Male 2) Female

2. Age: _____

3. Working day:

3.1 Total time at home: __ hour__ minute

3.1.1 Indoor time: __ hour__ minute

3.1.2 Outdoor time: __ hour__ minute

3.2 Total time at school: __ hour__ minute

3.2.1 Indoor time: __ hour__ minute

3.2.2 Outdoor time: __ hour__ minute

3.3 Total time at travel: __ hour__ minute

3.3.1 Indoor time: __ hour__ minute

3.3.2 Outdoor time: __ hour__ minute

3.4 Total time at other situations: __ hour__ minute

3.4.1 Indoor time: __ hour__ minute

3.4.2 Outdoor time: __ hour__ minute

4. Rest day:

4.1 Total time at home: __ hour__ minute

4.1.1 Indoor time: __ hour__ minute

4.1.2 Outdoor time: __ hour__ minute

4.2 Total time at school: __ hour__ minute

4.2.1 Indoor time: __ hour__ minute

4.2.2 Outdoor time: __ hour__ minute

4.3 Total time at travel: __ hour__ minute

4.3.1 Indoor time: __ hour__ minute

4.3.2 Outdoor time: __ hour__ minute

4.4 Total time at other situations: __ hour__ minute

4.4.1 Indoor time: __ hour__ minute

4.4.2 Outdoor time: __ hour__ minute

**References：**

1 Lee, M. L., Vassilaros, D. L. & Later, D. W. Capillary column gas chromatography of environmental polycyclic aromatic compounds. *International Journal of Environmental Analytical Chemistry* **11**, 251-262 (1982).

2 Sawicki, E., . Analysis for airborne particulate hydrocarbons: their relative proportions as affected by different types of pollution. *National Cancer Institute Monograph* **9**, 201-220 (1962).

3 Khalili, N. R., Scheff, P. A. & Holsen, T. M. PAH source fingerprints for coke ovens, diesel and, gasoline engines, highway tunnels, and wood combustion emissions. *Atmospheric Environment* **29**, 533-542 (1995).

4 Yingjun, C. *et al.* Emission factors for carbonaceous particles and polycyclic aromatic hydrocarbons from residential coal combustion in China. *Environmental Science & Technology* **39**, 1861-1867 (2005).

5 Zhao, X. G. & Duan, X. L. *Exposure factors handbook of Chinese population.Adults* (China Environment Science Press 2014).

6 Xia, Z. *et al.* Pollution level, inhalation exposure and lung cancer risk of ambient atmospheric polycyclic aromatic hydrocarbons (PAHs) in Taiyuan, China. *Environ Pollut* **173**, 150-156 (2013).
